# Oxonium 2-carb­oxy-3-(2-fur­yl)acrylate

**DOI:** 10.1107/S1600536809017760

**Published:** 2009-05-20

**Authors:** Wen-Xian Liang, Gang Wang, Zhi-Rong Qu

**Affiliations:** a Ordered Matter Science Research Center, College of Chemistry and Chemical Engineering, Southeast University, Nanjing 210096, People’s Republic of China

## Abstract

In the title compound, H_3_O^+^·C_8_H_5_O_5_^−^, neighbouring cations and anions are linked by O—H⋯O hydrogen bonds, forming a one-dimensional chain framework along [001]. The crystal structure is further stabilized by π–π inter­actions, with centroid–centroid distances of 3.734 (3) Å.

## Related literature

For the synthesis of β-amino­acids as precursors of novel biologically active compounds, see: O’Callaghan, *et al.* (1998 ▶); Cohen *et al.* (2002 ▶); Zeller *et al.* (1965 ▶).
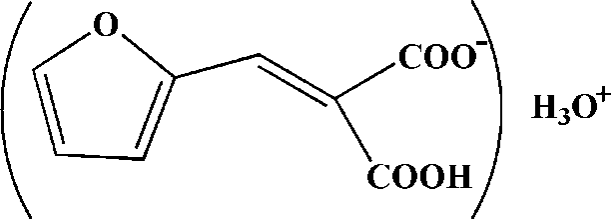


## Experimental

### 

#### Crystal data

H_3_O^+^·C_8_H_5_O_5_^−^
*M* *_r_* = 200.14
Monoclinic, 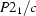

*a* = 13.664 (3) Å
*b* = 8.7518 (18) Å
*c* = 7.4664 (15) Å
β = 99.13 (3)°
*V* = 881.5 (3) Å^3^
*Z* = 4
Mo *K*α radiation
μ = 0.13 mm^−1^
*T* = 293 K
0.50 × 0.45 × 0.15 mm

#### Data collection

Rigaku SCXmini diffractometer
Absorption correction: multi-scan (*CrystalClear*; Rigaku, 2005 ▶) *T*_min_ = 0.935, *T*_max_ = 0.980
7954 measured reflections
1727 independent reflections
1406 reflections with *I* > 2σ(*I*)
*R*_int_ = 0.030

#### Refinement

*R*[*F* ^2^ > 2σ(*F* ^2^)] = 0.071
*wR*(*F* ^2^) = 0.218
*S* = 1.06
1727 reflections
128 parameters
H-atom parameters constrained
Δρ_max_ = 0.46 e Å^−3^
Δρ_min_ = −0.75 e Å^−3^

### 

Data collection: *CrystalClear* (Rigaku, 2005 ▶); cell refinement: *CrystalClear*; data reduction: *CrystalClear*; program(s) used to solve structure: *SHELXS97* (Sheldrick, 2008 ▶); program(s) used to refine structure: *SHELXL97* (Sheldrick, 2008 ▶); molecular graphics: *SHELXTL/PC* (Sheldrick, 2008 ▶); software used to prepare material for publication: *PRPKAPPA* (Ferguson, 1999 ▶).

## Supplementary Material

Crystal structure: contains datablocks I, global. DOI: 10.1107/S1600536809017760/rz2321sup1.cif

Structure factors: contains datablocks I. DOI: 10.1107/S1600536809017760/rz2321Isup2.hkl

Additional supplementary materials:  crystallographic information; 3D view; checkCIF report

## Figures and Tables

**Table 1:** Hydrogen-bond geometry (Å, °)

*D*—H⋯*A*	*D*—H	H⋯*A*	*D*⋯*A*	*D*—H⋯*A*
O1*W*—H1*WB*⋯O4^i^	0.85	2.58	3.044 (4)	115
O1*W*—H1*WA*⋯O3^i^	0.85	2.42	3.188 (4)	150
O1*W*—H1*WC*⋯O4^ii^	0.85	2.48	3.201 (4)	143
O2—H2*A*⋯O4^iii^	0.82	1.74	2.552 (3)	169

Symmetry codes: (i) 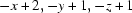; (ii) 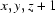; (iii) 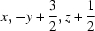.

**Fig. 1.**
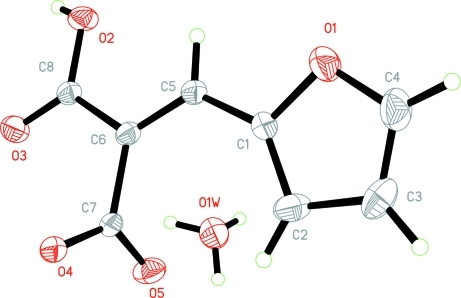
The molecular structure of the title compound with displacement ellipsoids drawn at the 30% probability level.

**Fig. 2.**
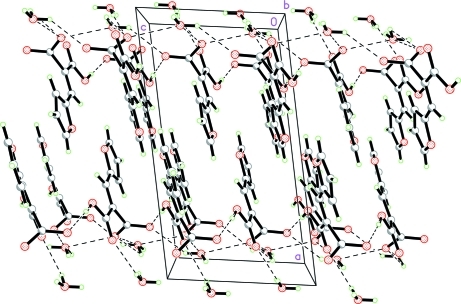
Packing diagram of the title compound viewed along the b axis. Hydrogen bonds are shown as dashed lines.

## supplementary crystallographic information

### Comment

2-[(Furan-2-yl)methylene]malonic acid is a important bicarboxylic acid widely used in coordination chemistry and as an intermediate product in the synthesis of β-aminoacids. Recently, there has been an increased interest in the enantiomeric preparation of β-aminoacids as precursors for the synthesis of novel biologically active compounds (O'Callaghan *et al.*, 1998; Cohen *et al.*, 2002; Zeller *et al.*, 1965). We report here the crystal structure of the title compound, which was prepared by the reaction of furan-2-carbaldehyde and malonic acid.

The asymmetric unit of the title compound (Fig.1) consists of a 2-[(furan-2-yl)methylene]malonate anion and a oxonium cation. The values of the C–O bond lengths in the carboxylic groups are consistent with a single bond character of the C8–O2 bond (1.308 (4)Å) and with a delocalized double bond character for the C7–O4 and C7–O5 bonds (1.262 (4) and 1.240 (4) Å, respectively). In the crystal packing (Fig. 2), classical intermolecular O—H···O hydrogen bonds connect neighbouring cations and anions, resulting in a one-dimensional chain framework along the *c* axis (Table 1). The crystal structure is further stabilized by π–π stacking interactions (Table 2) involving adjacent furane rings.

### Experimental

A mixture of furan-2-carbaldehyde (0.5 mol, 0.48 g) and malonic acid (0.5 mol, 0.52 g) in ethanol (20 ml) was added in a flask and refluxed for 24 h. The resulting precipitate was separated and dissolved in an ethanol/water (5:1 v/v). Colourless single crystals of the title compound suitable for X-ray analysis were obtained on slow evaporation of the solvents over a period of 48 h.

### Refinement

The H atoms bound to O atoms were located in a difference Fourier map and refined with O—H = 0.82-0.85 Å and *U*_iso_(H) = 1.5*U*_iso_(O). All other H atoms were placed geometrically and allowed, with C—H = 0.93 Å and *U*_iso_(H) = 1.2*U*_iso_(C).

### Crystal data

**Table:**

H_3_O^+^·C_8_H_5_O_5_^−^	*F*(000) = 416
*M**_r_* = 200.14	*D*_x_ = 1.508 Mg m^−^^3^
Monoclinic, *P*2_1_/*c*	Mo *K*α radiation, λ = 0.71073 Å
Hall symbol: -P 2ybc	Cell parameters from 1406 reflections
*a* = 13.664 (3) Å	θ = 3.1–27.4°
*b* = 8.7518 (18) Å	µ = 0.13 mm^−^^1^
*c* = 7.4664 (15) Å	*T* = 293 K
β = 99.13 (3)°	Prism, colourless
*V* = 881.5 (3) Å^3^	0.50 × 0.45 × 0.15 mm
*Z* = 4	

### Data collection

**Table:**

Rigaku SCXmini diffractometer	1727 independent reflections
Radiation source: fine-focus sealed tube	1406 reflections with *I* > 2σ(*I*)
graphite	*R*_int_ = 0.030
Detector resolution: 13.6612 pixels mm^-1^	θ_max_ = 26.0°, θ_min_ = 3.0°
CCD profile fitting scans	*h* = −16→16
Absorption correction: multi-scan (*CrystalClear*; Rigaku, 2005)	*k* = −10→10
*T*_min_ = 0.935, *T*_max_ = 0.980	*l* = −9→9
7954 measured reflections	

### Refinement

**Table:**

Refinement on *F*^2^	Secondary atom site location: difference Fourier map
Least-squares matrix: full	Hydrogen site location: inferred from neighbouring sites
*R*[*F*^2^ > 2σ(*F*^2^)] = 0.071	H-atom parameters constrained
*wR*(*F*^2^) = 0.218	*w* = 1/[σ^2^(*F*_o_^2^) + (0.1228*P*)^2^ + 1.0867*P*] where *P* = (*F*_o_^2^ + 2*F*_c_^2^)/3
*S* = 1.06	(Δ/σ)_max_ < 0.001
1727 reflections	Δρ_max_ = 0.46 e Å^−^^3^
128 parameters	Δρ_min_ = −0.74 e Å^−^^3^
0 restraints	Extinction correction: *SHELXL97* (Sheldrick, 2008)
Primary atom site location: structure-invariant direct methods	Extinction coefficient: 0.0014 (2)

### Special details

**Table:**

Geometry. All e.s.d.'s (except the e.s.d. in the dihedral angle between two l.s. planes) are estimated using the full covariance matrix. The cell e.s.d.'s are taken into account individually in the estimation of e.s.d.'s in distances, angles and torsion angles; correlations between e.s.d.'s in cell parameters are only used when they are defined by crystal symmetry. An approximate (isotropic) treatment of cell e.s.d.'s is used for estimating e.s.d.'s involving l.s. planes.
Refinement. Refinement of *F*^2^ against ALL reflections. The weighted *R*-factor *wR* and goodness of fit *S* are based on *F*^2^, conventional *R*-factors *R* are based on *F*, with *F* set to zero for negative *F*^2^. The threshold expression of *F*^2^ > σ(*F*^2^) is used only for calculating *R*-factors(gt) *etc*. and is not relevant to the choice of reflections for refinement. *R*-factors based on *F*^2^ are statistically about twice as large as those based on *F*, and *R*- factors based on ALL data will be even larger.

### Fractional atomic coordinates and isotropic or equivalent isotropic displacement parameters (Å^2^)

**Table:**

	*x*	*y*	*z*	*U*_iso_*/*U*_eq_	
C1	0.6364 (2)	0.3648 (4)	0.3879 (4)	0.0326 (7)	
C2	0.6705 (3)	0.2258 (4)	0.3518 (5)	0.0471 (9)	
H2	0.7334	0.2021	0.3275	0.057*	
C3	0.5905 (3)	0.1220 (4)	0.3582 (6)	0.0552 (10)	
H3	0.5918	0.0168	0.3408	0.066*	
C4	0.5149 (3)	0.2026 (5)	0.3932 (6)	0.0548 (10)	
H4	0.4530	0.1626	0.4032	0.066*	
C5	0.6780 (2)	0.5157 (3)	0.4112 (4)	0.0316 (7)	
H5	0.6393	0.5894	0.4562	0.038*	
C6	0.7666 (2)	0.5621 (3)	0.3751 (4)	0.0294 (7)	
C7	0.8392 (2)	0.4604 (3)	0.3007 (4)	0.0281 (7)	
C8	0.8008 (2)	0.7221 (3)	0.4080 (4)	0.0299 (7)	
O1	0.53944 (18)	0.3521 (3)	0.4129 (4)	0.0502 (7)	
O2	0.73994 (16)	0.8126 (3)	0.4760 (3)	0.0389 (6)	
H2A	0.7694	0.8902	0.5153	0.058*	
O3	0.88184 (17)	0.7637 (3)	0.3750 (3)	0.0436 (7)	
O4	0.84296 (16)	0.4683 (2)	0.1332 (3)	0.0354 (6)	
O5	0.89313 (18)	0.3763 (3)	0.4078 (3)	0.0431 (6)	
O1W	0.9457 (2)	0.4115 (3)	0.7823 (4)	0.0579 (8)	
H1WC	0.9463	0.4130	0.8963	0.087*	
H1WA	0.9797	0.3362	0.7549	0.087*	
H1WB	0.9703	0.4941	0.7495	0.087*	

### Atomic displacement parameters (Å^2^)

**Table:**

	*U*^11^	*U*^22^	*U*^33^	*U*^12^	*U*^13^	*U*^23^
C1	0.0310 (16)	0.0324 (16)	0.0356 (16)	−0.0014 (12)	0.0094 (12)	0.0014 (12)
C2	0.0446 (19)	0.0367 (19)	0.059 (2)	0.0089 (15)	0.0065 (16)	−0.0065 (16)
C3	0.068 (3)	0.0307 (18)	0.064 (2)	−0.0096 (18)	0.001 (2)	−0.0026 (17)
C4	0.050 (2)	0.048 (2)	0.067 (3)	−0.0196 (18)	0.0112 (18)	−0.0031 (19)
C5	0.0362 (17)	0.0269 (15)	0.0333 (15)	0.0027 (12)	0.0106 (12)	−0.0005 (12)
C6	0.0336 (16)	0.0230 (14)	0.0319 (15)	0.0023 (12)	0.0062 (12)	−0.0003 (11)
C7	0.0301 (15)	0.0205 (13)	0.0344 (16)	−0.0016 (11)	0.0074 (12)	0.0002 (11)
C8	0.0331 (16)	0.0250 (14)	0.0318 (15)	0.0008 (12)	0.0061 (12)	−0.0013 (12)
O1	0.0416 (14)	0.0451 (15)	0.0675 (17)	−0.0090 (11)	0.0199 (12)	−0.0047 (12)
O2	0.0382 (12)	0.0280 (11)	0.0521 (14)	0.0001 (9)	0.0120 (10)	−0.0121 (10)
O3	0.0409 (13)	0.0333 (12)	0.0602 (16)	−0.0059 (10)	0.0195 (11)	−0.0077 (11)
O4	0.0447 (13)	0.0290 (12)	0.0352 (12)	0.0061 (9)	0.0149 (9)	0.0025 (9)
O5	0.0481 (14)	0.0401 (13)	0.0397 (12)	0.0148 (11)	0.0030 (10)	0.0027 (10)
O1W	0.0586 (17)	0.0583 (17)	0.0589 (17)	−0.0026 (14)	0.0162 (13)	0.0010 (13)

### Geometric parameters (Å, °)

**Table:**

C1—C2	1.345 (5)	C6—C8	1.485 (4)
C1—O1	1.372 (4)	C6—C7	1.503 (4)
C1—C5	1.437 (4)	C7—O5	1.240 (4)
C2—C3	1.428 (6)	C7—O4	1.262 (4)
C2—H2	0.9300	C8—O3	1.226 (4)
C3—C4	1.311 (6)	C8—O2	1.308 (4)
C3—H3	0.9300	O2—H2A	0.8200
C4—O1	1.352 (5)	O1W—H1WC	0.8500
C4—H4	0.9300	O1W—H1WA	0.8500
C5—C6	1.344 (4)	O1W—H1WB	0.8500
C5—H5	0.9300		
			
C2—C1—O1	109.0 (3)	C5—C6—C8	121.5 (3)
C2—C1—C5	135.4 (3)	C5—C6—C7	124.3 (3)
O1—C1—C5	115.5 (3)	C8—C6—C7	114.3 (2)
C1—C2—C3	106.1 (3)	O5—C7—O4	123.9 (3)
C1—C2—H2	126.9	O5—C7—C6	118.2 (3)
C3—C2—H2	126.9	O4—C7—C6	117.8 (3)
C4—C3—C2	107.2 (3)	O3—C8—O2	123.2 (3)
C4—C3—H3	126.4	O3—C8—C6	121.1 (3)
C2—C3—H3	126.4	O2—C8—C6	115.6 (3)
C3—C4—O1	110.7 (3)	C4—O1—C1	107.0 (3)
C3—C4—H4	124.7	C8—O2—H2A	109.5
O1—C4—H4	124.7	H1WC—O1W—H1WA	109.5
C6—C5—C1	127.2 (3)	H1WC—O1W—H1WB	109.5
C6—C5—H5	116.4	H1WA—O1W—H1WB	109.5
C1—C5—H5	116.4		

### Hydrogen-bond geometry (Å, °)

**Table:**

*D*—H···*A*	*D*—H	H···*A*	*D*···*A*	*D*—H···*A*
O1W—H1WB···O4^i^	0.85	2.58	3.044 (4)	115
O1W—H1WA···O3^i^	0.85	2.42	3.188 (4)	150
O1W—H1WC···O4^ii^	0.85	2.48	3.201 (4)	143
O2—H2A···O4^iii^	0.82	1.74	2.552 (3)	169

Symmetry codes: (i) −*x*+2, −*y*+1, −*z*+1; (ii) *x*, *y*, *z*+1; (iii) *x*, −*y*+3/2, *z*+1/2.

### Table 2 π-π stacking interactions ( α is the dihedral angle between the planes, DCC is the length of the CC vector (centroid-to-centroid), τ is the angle subtended by the plane normal to CC. Cg1 is the centroid of the O1–C1/C4 ring)

**Table:**

Group 1	Group 2	α /°	DCC /Å	τ /°
Cg1	Cg1^i^	16.42	3.734 (3)	19.96

Symmetry code: (i) x, 1/2-y, -1/2+z
